# Recurrent Omental Hemangiopericytoma: A Therapeutic Challenge

**DOI:** 10.1155/2016/2075157

**Published:** 2016-03-21

**Authors:** Sara Jaber, Ira Winer, Nabila Rasool

**Affiliations:** ^1^Division of Gynecologic Oncology, Department of Women's Health Services, Henry Ford Hospital, Detroit, MI 48202, USA; ^2^Department of Oncology, Division of Gynecologic Oncology, Wayne State University, Karmanos Cancer Center, 4160 John R., Suite 721, Detroit, MI 48201, USA

## Abstract

Hemangiopericytomas are vascular tumors with a susceptibility to arise anywhere in the human body. We present a case of a 68-year-old female with primary omental hemangiopericytoma and a two-time recurrence managed with surgery and close follow-up. The first recurrence was at 52 months and the second at 37 months following the prior presentation. No adjuvant chemotherapy or radiation therapy was administered. Given the widespread nature of the cell of origin, routine follow-up postoperatively with interval imaging in order to detect recurrences is imperative. Pathologic tumor characteristics may determine potential for recurrence and may also assist in determining whether adjuvant treatment modalities should be included in the management plan. Review of the English literature reveals a total of 24 cases of omental hemangiopericytomas inclusive of the current report.

## 1. Introduction

Hemangiopericytomas are a rare form of vascular tumors that arise from the pericytes of Zimmerman that surround capillaries and function to regulate blood flow. They were first recognized by Schmidt in 1937 and fully described and named by Stout and Murray in 1942 [1]. Given the cell of origin, these vascular tumors can arise anywhere in the body. The most common sites reported are the lower limb musculature, pelvic fossa, retroperitoneum, and the head and neck region [2, 3]. Median age of onset is variable ranging from 45 to 55 years with an equal predilection for men and women [2, 3]. The classic pathologic appearance of a hemangiopericytoma is a well-encapsulated hypervascular tumor [4]. We present a case of primary omental hemangiopericytoma with subsequent local recurrences.

## 2. Case Report

**Figure 1:**
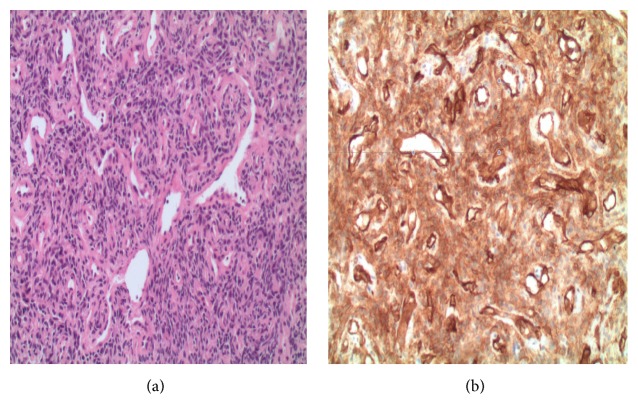
(a) Microscopic description: the tumor is cellular and composed of many variable ectatic and compressed thin walled branching vessels, giving staghorn configuration. The surrounding tumor cells are spindled to round with scant pale to eosinophilic cytoplasm, bland vesicular nuclei, and low mitotic activity. (b) Immunohistochemically, the tumor cells stain positive for CD34. The combined morphology and immunoprofile are consistent with hemangiopericytoma.

A 68-year-old G1P1001 Caucasian female with a known past medical history of hyperlipidemia, gastroesophageal reflux disease, and depression presented in 2006 with complaints of dull, achy pain located in the lower abdomen. She underwent endoscopic evaluation by her gastroenterologist with negative findings. As part of the work-up, she had a pelvic ultrasound, which demonstrated a large, solid heterogeneous mass superior to the ovaries measuring 15.4 × 14.7 × 8.6 cm with prominent internal blood flow. CA-125 (cancer antigen-125) was elevated at 77 U/mL with normal CEA (carcinoembryonic antigen), AFP (alpha-fetoprotein), and *β*HCG (beta-human chorionic gonadotropin). She underwent an exploratory laparotomy with lysis of adhesions and resection of the abdominal tumor with partial omentectomy because of concern for a primary ovarian carcinoma. Intraoperative findings included numerous dilated blood vessels in the omentum measuring up to 1.5 cm in diameter with a 25 × 20 cm soft, friable mass. The mass occupied most of the midabdomen, encasing the omentum, and was attached to the sigmoid mesentery and bladder peritoneum. The ovaries, uterus, and remaining pelvic structures were normal in appearance. There was no evidence of metastatic disease. The estimated blood loss (EBL) at the time of surgery was 1 liter and the patient received 2 units of packed red blood cells (PRBCs). Final pathology revealed “hemangiopericytoma of the abdomen with low malignant potential.” Histological findings showed a staghorn vascular pattern with solid perivascular areas formed by plump, comma-shaped cells with rare mitoses (less than 2 per 10 high power fields (HPF)) (Figure 1(a)). On immunohistochemical examination, tumor cells exhibited vimentin and CD34 positivity (a marker for vascular endothelial cells) with CD31 highlighting the vasculature (Figure 1(b)). S-100, keratin, and EMA were all negative; reticulin staining was positive surrounding the cords of cells. The patient did not receive any further adjuvant treatment and continued to follow up with her gynecologist oncologist.

She presented again to the gynecology oncology clinic in early 2011, 52 months after her primary surgery with symptoms of a “pinching sensation” and concerns for a palpable mass in the left lower quadrant of her abdomen of few months' duration. Computed tomography (CT) imaging of the abdomen and pelvis revealed interval development of a 2 × 1 cm hyperdense soft tissue lesion with a midline pelvic ventral hernia. Follow-up magnetic resonance imaging (MRI) showed a 2.1 × 1.5 cm ovoid-shaped mass probably originating in the peritoneal surface interposed between the lower uterine segment and sigmoid colon which demonstrated slightly hyperintense T2 signal in comparison to muscle with isointense T1 signal and uniform postcontrast enhancement. She underwent a second-exploratory laparotomy, tumor reductive surgery, total abdominal hysterectomy, bilateral salpingooophorectomy, segmental ileal resection with side-to-side reanastomosis, and repair of the ventral hernia. Intraoperative findings revealed rectosigmoid nodules as well as small nodules involving the ileum. Pathology was again consistent with hemangiopericytoma involving the resected rectosigmoid nodules, small bowel nodules with microscopic small bowel serosal residual disease. Histologic examination revealed uniform spindled to round cells with small amounts of pale or eosinophilic cytoplasm, indistinct margins, bland vesicular nuclei, and low mitotic activity. Numerous staghorn vascular spaces of varying sizes lined by flattened endothelium were also observed. Within the mass were alternating hypercellular perivascular areas formed by plump cells as well as hypocellular areas with increased collagen surrounding individual cells and occasional multinucleated giant cells. Immunohistochemical stains revealed CD 34 and focal CD 10 positivity. CD31, calretinin, HBME-1 (anti-mesothelioma antibody), C-kit, keratin, and S-100 all stained negative. Given findings consistent with recurrent hemangiopericytoma, the plan of care at the time included routine surveillance with no adjuvant chemotherapy/radiation therapy. She was followed up with imaging of the abdomen/pelvis.

Imaging performed 37 months after the last surgery showed a 2 cm enhancing solid mass in the left hemipelvis interposed between the proximal sigmoid serosa and adjacent small bowel and subjacent to the medial left urinary bladder. Another adjacent enlarging 1.7 × 1.9 cm enhancing mass with central necrosis or hypoattenuation was also seen just above the subjacent bladder and vaginal cuff. The patient elected to proceed forward with surgical resection and underwent a third-exploratory laparotomy, extensive lysis of adhesions, enterolysis, and tumor debulking. Intraoperative findings included a 3 cm ileal mesenteric mass with additional tumor nodules (4 cm rectosigmoid mesenteric mass, 3 cm mesenteric rectosigmoid mass, 3 cm epiploic mass adherent to mesentery of rectosigmoid, and 3 cm rectosigmoid mesenteric nodule adherent to bladder peritoneum). Pathology was again consistent with recurrent hemangiopericytoma. No adjuvant therapy was recommended at the time except close surveillance and imaging.

## 3. Discussion

Hemangiopericytomas have a distinct set of clinical, histopathological, and immunohistochemical features. These tumors are characterized by a unique staghorn appearance composed of thin walled vessels surrounded by ovoid and short spindle shaped cells [5, 6]. Immunohistochemical examination will reveal positivity for CD 34, a marker for platelet endothelial cells, as well as antismooth muscle actin, type IV collagen, and vimentin, and negativity for S-100 protein and cytokeratin [7]. In our patient, pathological evaluations of the primary and recurrent tumors were consistent with hemangiopericytoma.

Hemangiopericytomas in general have been found to have a favorable prognosis, despite the potential for recurrence and/or metastasis even after surgical management [8, 9]. Enzinger and Smith in 1976 examined 106 patients with hemangiopericytomas of which follow-up was available on 93 patients [4]. Of the patients who were alive (71), two had evidence of recurrent disease and four had exhibited metastatic disease versus thirteen with either recurrent or metastatic disease in the deceased group (22) [4]. The 10-year survival was reported as 70% [4]. A more recent study from MD Anderson published in 1998 found that 32% of primary hemangiopericytomas had local recurrences on follow-up with a 5-year actuarial survival of 71% [10]. Another study by Espat et al. in 2002 reported a 4% local recurrence rate and a 20% metastatic rate with 2- and 5-year survival rates of 93% and 86%, respectively [6]. Factors that may help distinguish between high-grade and low-grade lesions include cellularity, mitotic activity, and the presence of hemorrhage and necrosis [11]. Malignant hemangiopericytomas are described as those lesions with large tumor size (>5 cm), increased mitotic rate (≥4 mitoses per 10 HPF), high degree of cellularity, presence of immature and pleomorphic tumor cells, and foci of hemorrhage and necrosis [4]. McMaster et al. used histologic criteria to classify hemangiopericytomas as benign, borderline malignant, or malignant [9]. 37.5% of borderline and 78% of the malignant tumors exhibited metastasis [9]. Among the tumors with benign histology, the vascular pattern was rather prominent. Four of the twelve benign tumors that were examined exhibited slight anaplasia [9]. No mitotic figures were perceived in nine of these tumors while only three specimens exhibited 1 mitotic figure per 20 HPF [9]. On the other hand, all the borderline tumors demonstrated mitotic figures; the average was 1 mitotic figure per 20 HPF among seven of the sixteen tumors [9]. Tumors with 1 mitotic figure per 10 HPF and slight cellular anaplasia or 1 mitotic figure per 20 HPF and moderate cellular anaplasia were categorized with malignant potential [9]. The histological features of our reported omental hemangiopericytoma exhibited benign features except for a gross tumor size of >5 cm which is a feature of malignant behavior. Despite that, it was best described as a “low malignant potential tumor.”

Omental hemangiopericytomas represent around 10% of all primary solid omental tumors [12]. A review of the English literature revealed 24 cases of omental hemangiopericytoma inclusive of our patient. The interval to disease recurrence is relatively delayed with a median duration between primary diagnosis and the first recurrence estimated at 17 months (range: 1 month to greater than 7 years) [4]. Other reports have projected the median time to recurrence at 29 months [10]. When compared to other soft tissue sarcomas, hemangiopericytomas were noted to have a longer latency period prior to the first recurrence [8, 10]. Slupski et al. reported a recurrence of primary omental hemangiopericytoma occurring 18 years following primary resection [13]. Distant sites of metastasis include lung, liver, brain, scalp, chest wall, gastrointestinal tract, bone, orbit, vulva, and lymph nodes [9, 14]. The first recurrence in our case occurred 52 months after the primary surgery while the second recurrence ensued 37 months after the second-surgical resection, typical of this type of lesion.

Surgery with curative intent remains the primary mode of management of hemangiopericytomas arising from the omentum [15]. Performance of a complete omentectomy does not guarantee superior prognosis. Even in those cases in the literature wherein a total omentectomy was performed, recurrence was often found [12, 16, 17]. Metastatic and recurrent tumors may benefit from chemotherapy; however, there are no established chemotherapeutic regimens. Additionally, the slow progression of these cancers impacts the ability to discern true benefit from chemotherapy in terms of recurrence-free and overall survival [15]. Agents such as Doxorubicin and Adriamycin used solely or in combination with other chemotherapeutic drugs have shown good response [16, 18]. Sunitinib, a vascular endothelial growth factor receptor inhibitor and a platelet-derived growth factor receptor kinase inhibitor, has also shown favorable clinical benefit [19]. Other pharmaceutical agents such as pazopanib, a tyrosine-kinase inhibitor, have exhibited clinical efficacy and the potential for management of metastatic hemangiopericytomas; however more studies still need to be conducted [20]. Reports regarding radiation therapy on the other hand have been inconsistent [16, 21–24]. Our case was managed surgically with complete resection of the primary tumor and each of the two successive recurrences. In the interim, patient was followed up closely both clinically and with serial imaging. The decision not to give chemotherapy or radiotherapy in our case despite two local recurrences was based on multiple considerations including the ability to successfully resect the lesions, the size, histological appearance of these recurrences, and the indolent course of this disease. Hemangiopericytomas create a major postoperative challenge for the provider due to the increased potential for recurrence, the uncertain timeframe for the disease progression, and the absence of clear tools to detect recurrences. Some studies have suggested that a yearly PET scan may exemplify a valid tool for the ascertainment of disease recurrence [14].

## 4. Conclusion

Hemangiopericytomas have a high five-year overall survival; however, given the cellular origin of these tumors, long-term follow-up is essential as recurrences have been reported even after extended disease-free intervals. Predictors of recurrence and metastasis include histological appearance and behavior of the primary tumor. Surgery remains the chief method of therapy, with the potential for utilization of chemotherapy and/or radiation therapy as adjuvants in the more aggressive malignant cases with an unclear benefit to its use given the nature of the disease.
